# Are contributory causes of death in part 2 of the death certificate mediators of chains of morbid events leading to death?

**DOI:** 10.1186/s12963-025-00394-w

**Published:** 2025-08-07

**Authors:** Elizabet Ukolova

**Affiliations:** 1 Interdisciplinary Centre on Population Dynamics, University of Southern Denmark, Odense, Denmark; 2 Faculty of Science, Department of Demography and Geodemography, Charles University, Prague, Czechia

**Keywords:** Multiple causes of death, Contributory causes, Causal pies, Mediation

## Abstract

**Background:**

In the United States, over half of all deaths are attributed to five leading underlying causes of death (at the ICD-3 digit level). However, these underlying causes represent only 25% of the total medical information documented on death certificates. While previous studies have investigated associations between causes of death, none have specifically examined the mechanisms of interaction among these causes. This study aims to explore the role of contributory causes of death recorded in Part 2 of the death certificate in the lethal process.

**Methods:**

Working with U.S. Multiple Cause of Death Microdata in 2019, we use causal pie models to model the synergy between multiple causes of death.

**Results:**

The findings show how contributory causes in Part 2 affect the sequence of morbid events leading to death. Three broad categories of roles can be distinguished: (i) some contributory causes act as mediators in the chain of morbid events, (ii) others do not exhibit any interaction with the conditions listed in Part 1, and (iii) some might play a role in the development of underlying causes.

**Conclusion:**

Contributory causes listed in Part 2 play a crucial role in transitions to terminal morbid states. There is evidence that these are more than just conditions without a direct relationship to the underlying cause of death.

**Supplementary Information:**

The online version contains supplementary material available at 10.1186/s12963-025-00394-w.

## Introduction

Available data indicate that people typically die from more than just a single cause. However, the currently established approach to cause specific analysis of mortality relies on the automated selection of a single underlying cause of death, defined as the condition that initiated the sequence of events leading to death [1]. This approach offers a key advantage: it produces internationally comparable cause of death statistics, as the process for selecting the underlying cause of death (UCD) is standardized, at least in countries using the same software for automated coding of causes of death. On the other hand, this method inevitably results in a substantial loss of medical information. For instance, in the United States, sole focus on the underlying cause of death means ignoring nearly 70% of the diagnoses or conditions present at the time of death (Author's calculations based on [2]) and in Czechia, nearly 74% are disregarded (Author's calculations based on [3]). Among the most overlooked contributory causes of death in both the U.S. and Czechia are conditions from the cardiovascular and respiratory disease groups, as well as diabetes and sepsis [2, 3].

As a result, deaths arising from entirely different etiologies may appear to be caused by the same disease, which can lead to misunderstanding of the burden of individual diseases. This has been previously identified, for example, in diabetes [4–6], Alzheimer’s disease and dementia [7–9], sepsis [10–13], or heart diseases [14, 15]. These conditions are often classified as contributory causes of death (CC) and ultimately do not appear as the UCD in cause of death statistics.

The multiple cause of death (MCD) approach, on the other hand, uses all the information recorded on the death certificate, including both the UCD and CC. The CC are either listed in Part 1 or in Part 2 of the death certificate. According to international guidelines for recording causes of death, physicians should document the sequence of morbid events leading directly to death in Part 1 [16, 17]. Other significant conditions that contributed to the death but were not part of this sequence should be recorded in Part 2 [1, 18, 19]. Interest in these various types of MCD has grown remarkably over the past decade [20], largely due to the increasing prevalence of chronic diseases associated with population aging, which then translates into more complex processes of death [21]. However, the interactions between MCD have largely remained unexplored. Instead, prior research has primarily concentrated on exploratory analyses of MCD data [22].

In this study, we aim to address a simple question: Do contributory causes of death recorded in Part 2 of the death certificate mediate the effect of underlying cause on its consequent causes? According to the definition cited above, contributory causes in Part 2 should not have an etiological relationship to the causes reported in Part 1, as they neither originate from these causes nor do the causes in Part 1 originate from the contributory causes in Part 2. In other words, contributory causes in Part 2 are diseases that may elevate the risk of death without necessarily being the “reason for death” themselves. If a contributory cause is not found to act as a mediator within the causal chain, it can be regarded as a comorbidity present at the time of death rather than a disease essential for the progression of the chain toward the terminal morbid state.

Mediation is defined as the process that explains the relationship between an exposure and an outcome [23]. A mediator acts as an intermediate step in the causal pathway. Typically, structural equation modelling is employed to address questions of mediation [24, 25]. In the context where the objective is to model a mechanism involving only three factors (the CC recorded in Part 2, the underlying cause, and its consequent condition), this task can be formalized within the causal pie framework with mediators. Causal pie models, or sufficient component cause models, have been widely used in epidemiologic research to elucidate the complex interactive nature in outcome causation [25–30].

The rise of causal pie models has coincided with the increasing significance of chronic diseases, which are seldom, if ever, attributable to a single exposure [31]. Instead, their etiology is shaped by the interplay of multiple factors that form a causal web. Naturally, these developments have been reflected in mortality, which has increasingly been driven by chronic and degenerative diseases as well [16, 32, 33]. Since such diseases do not predominantly lead to immediate death [16, 32, 33], a gap has emerged between the onset of health decline and the time of death. This period is characterized by the gradual accumulation of diseases [34–36], which has sparked discussions about the nature of the dying process. Traditionally, cause-specific mortality is viewed as a competing risk problem, where accumulated diseases"compete"for an individual's life, and ultimately, only one disease"wins"[37, 38]. Alternatively, death can result from the interplay among these accumulated diseases [28, 39, 40], which aligns with the logic of causal pie models. According to this concept, each disease serves as a component of the pie, and it is the presence of all diseases that ultimately determines the timing of the outcome [26, 31, 39].

Here we apply causal pie models in MCD setting as follows.

In accordance with international rules for cause of death certification “death” is the terminating state in a chain of morbid events and it can be preceded by non-UCD recorded in Part 1, which, at the same time, is a consequence of the automatically selected UCD. The relationship between these morbid states may be mediated by the CC. If this were not the case, we might hypothetically expect that disrupting the morbid chain would prevent “death”. Focusing on CC, we aim to explore the nature of the interactions between automatically selected UCD, its consequent condition and CC recorded in Part 2, which is crucial for understanding the mechanism of the lethal process.

### Data

We work with US Multiple Cause-of-Death Mortality data from the most recent pre-COVID year available at the time of analysis (2019), that was obtained from the National Bureau of Economic Research [41], which compiles microdata on mortality from the National Vital Statistics System of the National Centre for Health Statistics (NCHS) [2]. Each record in the microdata is based on information extracted from death certificates. As previously mentioned, there are international guidelines for handling death certificate data. Once a physician completes the death certificate, the NCHS uses an Automated Coding System to convert medical terms into ICD codes and determine the underlying cause of death. This process relies on ACME software, which employs decision tables covering all possible relationships between diseases and it is responsible for selecting the underlying cause of death [1, 42]. At the end, two types of variables with multiple causes of death are generated. The first type, known as"Entity Axis Codes"(EAC), is later recoded into the second type,"Record Axis Codes"(RAC), in order to remove inconsistencies, such as redundant codes or diagnoses incompatible with the deceased person’s sex [42]. During the conversion from EAC to RAC, the information about exact placement of conditions on the death certificate is lost. Since the current analysis requires distinguishing between different types of causes of death, we rely on EAC. From them, we create triads of diseases, differentiating between conditions recorded in the Part II of the death certificate, the ACME-selected UCD, and its joint consequent condition recorded in the Part I.

However, criticism has been raised regarding the accuracy of the data indicating the sequence of conditions listed by the physician in Part I of the death certificate for inferring causality [43]. To address this, we first examined the reliability of the causal relationship between automatically selected UCDs and their consequent conditions, which we derived from EAC data. For that purpose, we used Iris version 5.8.4, a software for selecting the UCD that is utilized in most European countries, as well as in Canada, Australia, the Philippines, and others. We identified the most frequent combinations of causes of death on 3-digit level of ICD-10 classification, including their positions on the death certificate, and imported this data into the Iris software. The goal was to validate the causal relationship between the UCD and other conditions listed in Part 1, supporting the distinction between the UCD and its consequent condition in the causal pie models. The triads of diseases ultimately selected for analysis, are provided in Table 1 and their corresponding ICD-10 codes can be found in the Supplementary Material in Table S1. These triads consist of automatically selected UCD and its consequent condition, which are validated by Iris and appear most frequently with the leading CC.

**Table 1:** Selected triads of causes of death, by sex and age and frequency (‰)

UCD	CC in Part 2	non-UCD in Part 1	‰
*Males, 60–79 years*			
Acute myocardial infarction	Essential (primary) hypertension	Chronic ischaemic heart disease	3.7
Chronic ischaemic heart disease	Diabetes mellitus	Hypertensive heart disease	3.4
Chronic ischaemic heart disease	Essential (primary) hypertension	Cardiac arrest	3.2
Chronic ischaemic heart disease	Chronic kidney disease	Congestive heart failure	2.9
Chronic ischaemic heart disease	Essential (primary) hypertension	Congestive heart failure	2.7
Chronic ischaemic heart disease	Chronic obstructive pulmonary disease	Congestive heart failure	2.7
Acute myocardial infarction	Diabetes mellitus	Chronic ischaemic heart disease	2.4
Chronic ischaemic heart disease	Diabetes mellitus	Congestive heart failure	2.3
Chronic ischaemic heart disease	Diabetes mellitus	Cardiac arrest	2.1
Chronic ischaemic heart disease	Atrial fibrillation and flutter	Congestive heart failure	2.1
*Males, 80 years and over*			
Chronic ischaemic heart disease	Atrial fibrillation and flutter	Congestive heart failure	8.6
Chronic ischaemic heart disease	Chronic kidney disease	Congestive heart failure	6.3
Chronic ischaemic heart disease	Essential (primary) hypertension	Congestive heart failure	5.9
Chronic ischaemic heart disease	Chronic obstructive pulmonary disease	Congestive heart failure	4.0
Chronic ischaemic heart disease	Essential (primary) hypertension	Cardiac arrest	3.8
Acute myocardial infarction	Essential (primary) hypertension	Chronic ischaemic heart disease	3.1
Chronic ischaemic heart disease	Diabetes mellitus	Congestive heart failure	2.5
Chronic ischaemic heart disease	Atrial fibrillation and flutter	Cardiac arrest	2.4
Chronic ischaemic heart disease	Dementia	Congestive heart failure	2.2
Chronic obstructive pulmonary disease	Congestive heart failure	Respiratory failure	2.1
*Females, 60–79 years*			
Chronic obstructive pulmonary disease	Essential (primary) hypertension	Respiratory failure	2.5
Chronic obstructive pulmonary disease	Congestive heart failure	Respiratory failure	2.3
Acute myocardial infarction	Essential (primary) hypertension	Chronic ischaemic heart disease	2.2
Chronic ischaemic heart disease	Essential (primary) hypertension	Cardiac arrest	2.1
Chronic ischaemic heart disease	Diabetes mellitus	Hypertensive heart disease	1.9
Chronic ischaemic heart disease	Chronic obstructive pulmonary disease	Congestive heart failure	1.7
Chronic ischaemic heart disease	Essential (primary) hypertension	Congestive heart failure	1.7
Chronic ischaemic heart disease	Chronic kidney disease	Congestive heart failure	1.7
Chronic ischaemic heart disease	Diabetes mellitus	Congestive heart failure	1.5
Acute myocardial infarction	Diabetes mellitus	Chronic ischaemic heart disease	1.5
*Females, 80 years and over*			
Chronic ischaemic heart disease	Essential (primary) hypertension	Congestive heart failure	3.9
Chronic ischaemic heart disease	Atrial fibrillation and flutter	Congestive heart failure	3.5
Chronic ischaemic heart disease	Chronic kidney disease	Congestive heart failure	3.1
Chronic ischaemic heart disease	Essential (primary) hypertension	Cardiac arrest	2.6
Acute myocardial infarction	Essential (primary) hypertension	Chronic ischaemic heart disease	2.1
Chronic ischaemic heart disease	Chronic obstructive pulmonary disease	Congestive heart failure	2.1
Atrial fibrillation and flutter	Essential (primary) hypertension	Congestive heart failure	2.0
Chronic ischaemic heart disease	Dementia	Cardiac arrest	1.9
Chronic ischaemic heart disease	Dementia	Congestive heart failure	1.9
Chronic obstructive pulmonary disease	Congestive heart failure	Respiratory failure	1.9

Source: Author

The analysis is conducted separately by sex and age, distinguishing between two groups: those aged 60–79 and those aged 80 and older.

## Method

**Fig. 1:**
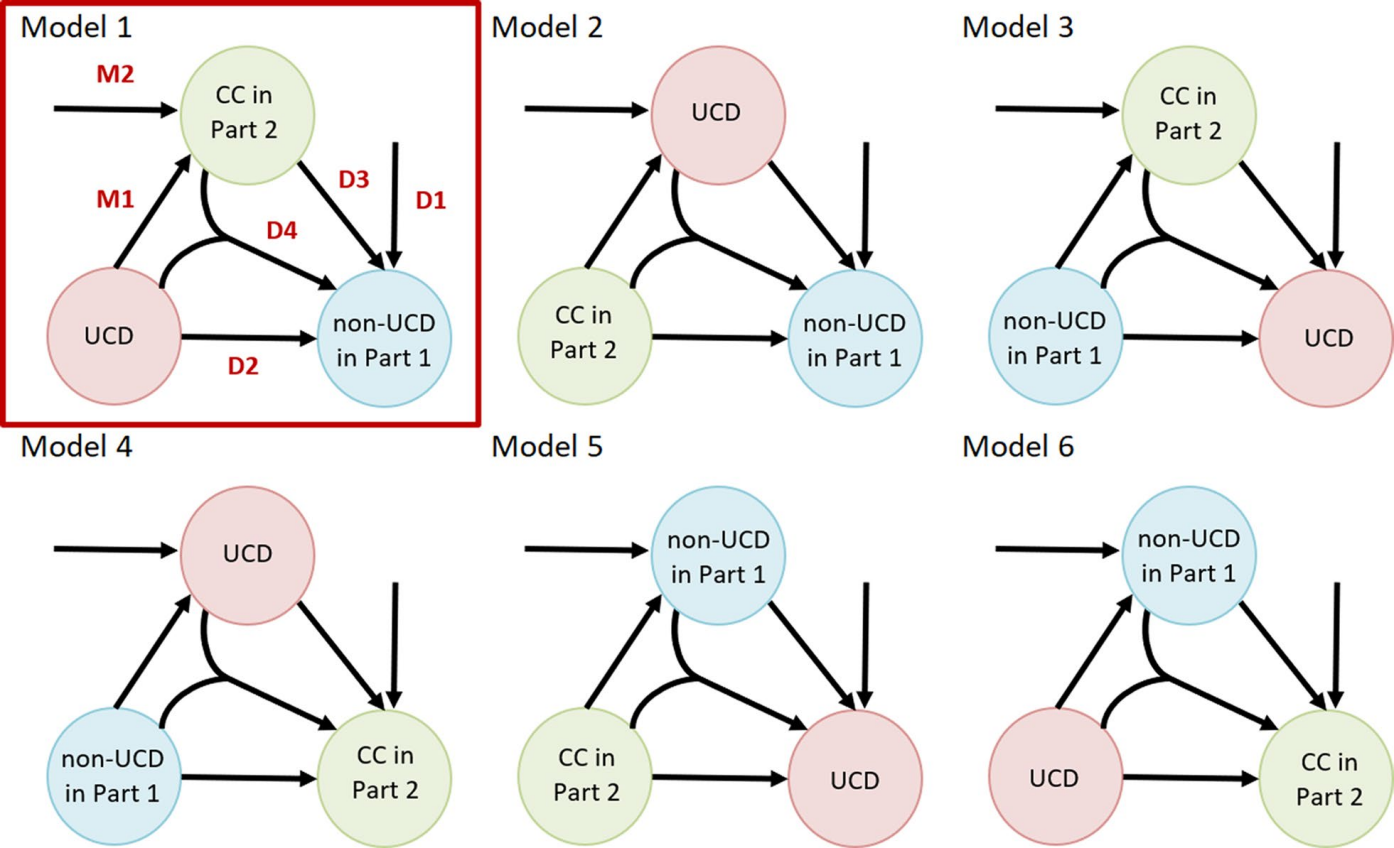
Causal pie models with mediators for different architecture of relations within triads of multiple causes of death. *Note* UCD = automatically selected underlying cause of death; non-UCD in Part 1 = non-underlying condition recorded in Part 1 of the death certificate, for which the “due to” relationship with UCD was checked with Iris software; CC in Part 2 = contributory cause of death recorded in Part 2 of the death certificate. The arrows represent all possible interplay scenarios between causes of death within the triads. *Source* Author

After the triads of diseases were selected, we employed six causal pie models, each with a different architecture representing the relationships between diseases, as illustrated in Fig. 1. Using these models in a complementary way allows us to move beyond pre-established relations between diseases, as described previously. Each model varies in the synergy it assumes between diseases. For instance, the Model 1 assumes alignment between WHO guidelines and how the death certifier completed the death certificate. This is because, in Model 1, the UCD is assumed to cause a non-UCD condition listed in Part 1 of the death certificate, with a CC listed in Part 2 potentially mediating this relationship, depending on the estimated model parameters. If no mediation occurs, the transition rate along the edge labelled as D2 in Fig. 1 would dominate. In contrast, if the CC is a necessary component in the transition between the UCD and its consequence, the transition along the edge D3 would become the most prominent in the model. In contrast to Model 1, Model 2 does not treat the CC recorded in Part 2 as a mediator in the chain of morbid events leading to death. In Model 2, the CC is considered a condition that potentially initiates the progression toward two other conditions: both the UCD and its consequent condition. This distinction is reflected in the direction of the arrows in the diagram for Model 2, as shown in Fig. 1. Model 3, on the other hand, represents a scenario where the relationship between the UCD and the non-UCD listed in Part 1 could be reversed from what would typically be expected under WHO guidelines for death certification. In this case, the flow along the edge labelled D2 would be the strongest, indicating a reversed causal relationship according to death certificate records.

As previously mentioned, mediation is defined as the process that explains the relationship between an exposure and an outcome [23]. Hafeman in [30] was the first to use causal pie models, also known as sufficient component cause models, to describe mediation. In this study, we follow his methodology and use macros written by Chen and Lee [44] to calculate the parameters of the causal pie models.

**Fig. 2:**
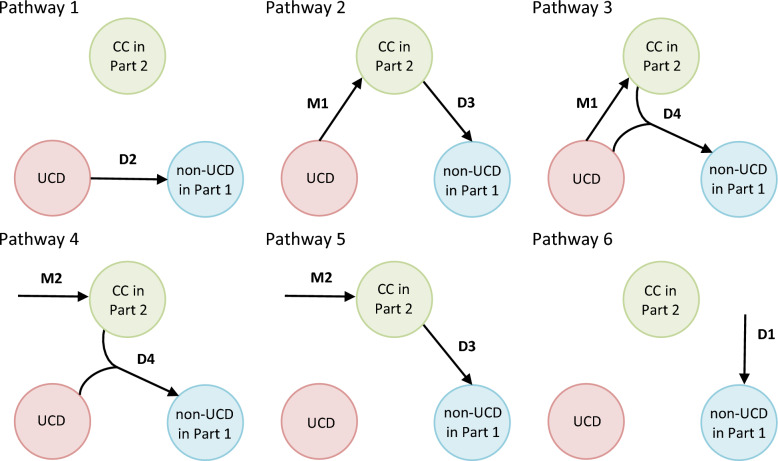
The pathways toward a terminating morbid state before death, Model 1. *Source* Adopted from [44, 45]

Figure 1 above illustrates the directed connections between UCD, non-UCD, and CC in Part 2. In each model, two stages of mediation can be identified: the M-stage and the D-stage. The M-stage represents the phase where the mediator is acquired, while the D-stage refers to the causation of the outcome [30, 44]. During the M-stage, the process progresses toward the mediator, which can be reached either through exposure (M1) or via factors outside the model (M2). Following this, the process moves toward the outcome, with four potential pathways: (i) from external factors not included in the model (D1), (ii) directly from the exposure (D2), (iii) via the mediator (D3), (iv) from both the mediator and the exposure (D4). Consequently, there are six potential pathways in each Model an individual may take to reach a terminal morbid state before death. In theory, this should correspond to the non-UCD recorded in Part 1, assuming accurate death certification. In Model 1, which represents this situation, each of these pathways is described as follows: [44, 45] (Fig. 2):

I. The UCD causes the non-UCD directly (path D2).
II. The UCD causes the CC, which in turn causes the non-UCD (path M1D3).
III. The UCD causes the CC, and then both interact to cause the non-UCD (path M1D4).
IV. The UCD and an exogenous mediator interact to cause the non-UCD (path M2D4).
V. An exogenous mediator causes the non-UCD directly (path M2D3).
VI. Neither the UCD nor the CC causes the non-UCD (path D1).

In pathways IV and V, the term'exogenous'implies that the mediator (in this case, the CC in Part 2) is not caused by the UCD; rather, its development is influenced by factors outside of the modelled mechanism. Figure 2 illustrates each of the situations described in pathways I–VI for Model 1. For the remaining models, explanations of the pathways are provided in the Supplementary Material. The application of all Models 1–6 comprehensively describes all possible relationships between the causes of death listed on the death certificate, rather than being limited to the synergy implied by assumed relationships between the UCD and non-UCD in Part 1, and the CC in Part 2. For example, the architecture of causal web in Models 4 and 6 allows for the modelling of cases where the CC from Part 2 arises due to an interaction between the UCD and non-UCD in Part 1. Similarly, Model 2 captures mechanisms where the CC in Part 2 contributes to the emergence of the UCD and serves as a necessary component for the transition to non-UCD in Part 1.

In conclusion, all six pathways are present in each model; however, their interpretation varies depending on the causal web that is different for each model (see Supplementary Material).

**Fig. 3:**
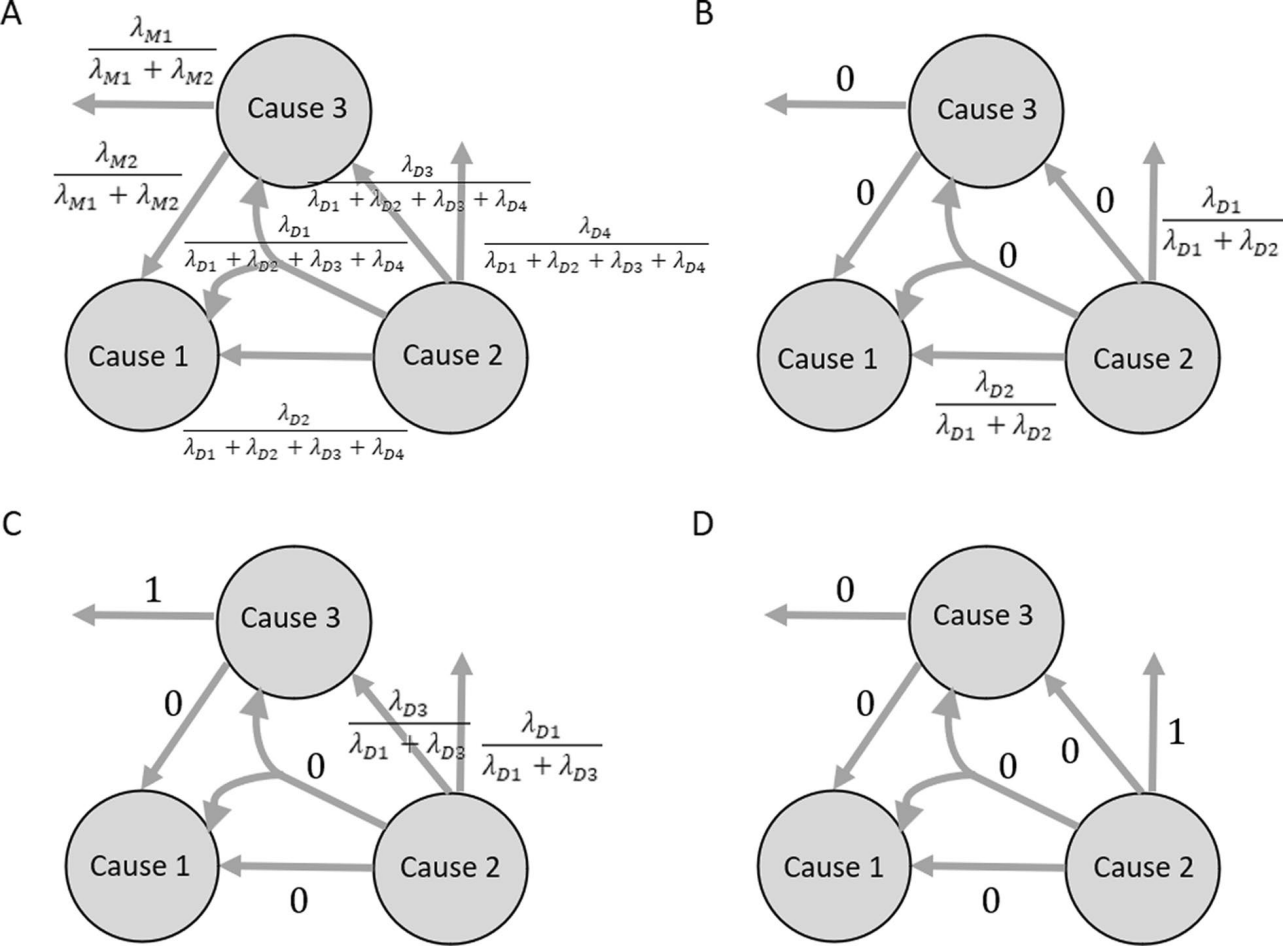
Attribution Process and Formulas for Attributable Fractions. *Source* Adopted from [44]

Several assumptions must be stated explicitly. First, we assume that UCD or CC in Part 2 do not act as protective factors against non-UCD in Part 1. In other words, we adopt the positive monotonicity assumption, consistent with other studies using the causal pie models [26, 46]. Second, we assume no redundancy, meaning that at the same time, there can be at most one arrival event. This property is essential for the attribution process described below. For example, in a population with all three diseases in Model 1 (Fig. 3, panel A), acquiring the CC recorded in Part 2 may occur either due to external factors or the UCD, but not both. This is because formulas used to calculate attributional fractions (Fig. 3) distribute the arrival rates for M1 and M2 proportionally across both these edges [44]. Third, we assume that the time interval between developing the diseases within the triads is irrelevant, meaning we set T = 1 in all the formulas presented below. This follows the approach used in studies by Chen and Lee in [44] and Hafeman in [30], where exact timing of onset of diseases was also unavailable.

With these assumptions in mind, we can proceed with the estimation of the causal pie parameters and attributable fractions. Causal pie parameters are arrival rates that represent the magnitude of flow along each pathway [44]. Attributable fractions, on the other hand, assign a probability to each pathway depicted in Fig. 2. In other words, attributable fractions are probabilities, that the synergy or interaction between the causes of death can be explained by each specific pathway [26]. These probabilities can be calculated for all subpopulations based on the presence or absence of any disease within the triads. However, our focus here is solely on interpreting the results within the population affected by all three diseases.

Suppose we have death counts by causes aggregated as shown in Table 2 and we can use them to calculate the corresponding raw estimates of the arrival rates. For this purpose, following set of equations is used [44]:

1 $${\lambda }_{M1}=-\frac{1}{T}\times ln\left(1-\frac{{m}_{2}+{m}_{4}}{{m}_{1}+{m}_{2}+{m}_{3}+{m}_{4}}\right)$$

2 $${\lambda }_{M2}=-\frac{1}{T}\times ln\left(1-\frac{{n}_{2}+{n}_{4}}{{n}_{1}+{n}_{2}+{n}_{3}+{n}_{4}}\right)-{\lambda }_{M1}$$

3 $${\lambda }_{D1}=-\frac{1}{T}\times ln\left(1-\frac{{m}_{3}}{{m}_{1}+{m}_{2}+{m}_{3}+{m}_{4}}\right)$$

4 $${\lambda }_{D2}=-\frac{1}{T}\times ln\left(1-\frac{{n}_{3}}{{n}_{1}+{n}_{2}+{n}_{3}+{n}_{4}}\right)-{\lambda }_{D1}$$

5 $${\lambda }_{D3}=\frac{{m}_{4}}{({m}_{2}+{m}_{4})\times (T-ET1)}-{\lambda }_{D1}$$

6 $${\lambda }_{D3}=\frac{{n}_{4}}{({n}_{2}+{n}_{4})\times (T-ET2)}-{\lambda }_{D1}-{\lambda }_{D2}-{\lambda }_{D3}$$

where *ET1* equals to:

**Table 2:** Distribution of deaths by UCD, non-UCD and CC status for each of the six causal pie models

Model 1				Model 2				Model 3			
M	D	E		M	D	E		M	D	E	
CC	non-UCD	UCD = 0	UCD = 1	UCD	non-UCD	CC = 0	CC = 1	CC	UCD	non-UCD = 0	non-UCD = 1
0	0	m1	n1	0	0	m1	m2	0	0	m1	m3
1	0	m2	n2	1	0	n1	n2	1	0	m2	m4
0	1	m3	n3	0	1	m3	m4	0	1	n1	n3
1	1	m4	n4	1	1	n3	n4	1	1	n2	n4
Model 4				Model 5				Model 6			
M	D	E		M	D	E		M	D	E	
UCD	CC	non-UCD = 0	non-UCD = 1	non-UCD	UCD	CC = 0	CC = 1	non-UCD	CC	UCD = 0	UCD = 1
0	0	m1	m3	0	0	m1	m2	0	0	m1	m2
1	0	n1	n3	1	0	m3	m4	1	0	m3	m4
0	1	m2	m4	0	1	n1	n2	0	1	n1	n2
1	1	n2	n4	1	1	n3	n4	1	1	n3	n4

Source: Author

M = mediator, E = exposure, D = terminal morbid state/outcome; see Supplementary Material for an example

7 $$ET1=\frac{\frac{1}{{\lambda }_{M1}}-(T+\frac{1}{{\lambda }_{M1}})\times {e}^{-{\lambda }_{M1}\times T}}{1-{e}^{-{\lambda }_{M1}\times T}}$$

And *ET2* equals to:

8 $$ET2=\frac{\frac{1}{{\lambda }_{M1}+{\lambda }_{M2}}-(T+\frac{1}{{\lambda }_{M1}+{\lambda }_{M2}})\times {e}^{-({\lambda }_{M1}+{\lambda }_{M2})\times T}}{1-{e}^{-({\lambda }_{M1}+{\lambda }_{M2})\times T}}$$

In Eqs. (1)–(8), m_i_ and n_i_ represent death counts corresponding to the fields of the contingency tables (Table 2), which classify deaths by the presence or absence of UCD, non-UCD, and CC in Part 2. For instance, n_1_ represents the number of deaths where neither a consequent condition of the disease originating chain of morbid events nor the mediator was acquired. In Model 1, this equals to number of deaths with UCD but without CC and non-UCD. However, in Model 2, this corresponds to m_2_, because the causal web in Model 2 is structured such that the UCD may (depending on the estimated parameters) mediate the relationship between CC in Part 2 and non-UCD (Fig. 2, Model 2). In the Supplementary Material in Table S2, we provide an example of Table 2 filled with the actual death counts for one of the triads we analyze.

In Eqs. (1)–(8), T denotes time, which is assumed to be equal to 1, as no information is available regarding the time elapsed between the onset of the diseases.

The raw estimates of arrival rates are then used as the starting point for the Newton–Raphson algorithm, which is used to find the optimal solution for their maximum likelihood. Details of this process are provided in the Supplementary Material. Here, we focus on how these arrival rates are applied in estimating the attributable fractions.

The"attribution"is a reverse process, in which the flows are used to calculate the probability of following each of the six pathways shown in Fig. 2. The attribution process is schematically illustrated in Fig. 3 by the reverse direction of arrows compared to Figs. 1 and 2. Each panel in Fig. 3 presents, along the arrows, the formulas that are further used to calculate the attributable fractions for different populations: (i) those with the complete set of diseases (Fig. 3, Panel A), (ii) those with diseases C1 and C2 (Fig. 3, Panel B), (iii) those with diseases C2​ and C3​ (Fig. 3, Panel C), and (iv) those with only disease C3 (Fig. 3, Panel D). For instance, in deaths where the death certificate lists only C3, the disease C3 must have been acquired through a mechanism outside the modelled triad. Therefore, the attributable fraction of path D1 is equal to 1 (Fig. 3, Panel D). Conversely, if the death involved both C3 and C2, the C3 could have been acquired either through external mechanisms or via C2. In this case (Fig. 3, Panel C), the attributable fraction is proportionally divided between paths D1 and D3.

The formulas shown in Fig. 3 are then combined with the share of deaths by cause combination to calculate the attributable fractions. Attributable fractions (AF) represent the probabilities that, given a specific set of diseases, the lethal process follows any of the pathways I-VI described in Fig. 2 and in the Supplementary Material. AFs are also calculated separately for each Model 1–6. The formulas used for these calculations are provided in the Supplementary Material. The authors of this computational procedure [44] highlight that their approach is advantageous because it guarantees that the attributable fractions always sum to one. This ensures logical consistency across various scenarios of synergy between diseases, as the sum of all probabilities of all possible pathways is always equal to 1.

The formulas presented in Fig. 3 clearly show that the resulting attributable fractions will differ depending on whether a person died with both the UCD and CC in Part 2, only one of them, or neither. As mentioned previously, the results presented below focus on the population with the complete set of diseases within the triads, as the primary aim of the paper is to examine the role of the CC in relation to the chain of morbid events leading to death in Part 1 of the death certificate.

## Results

### Study population

In 2019, there were 2.3 million deaths aged 60 years and over in the USA, with half of them being female. Of these deaths, 47% occurred in the 60–79 age group. In this younger age group, males accounted for a higher proportion of deaths than females (57% vs. 43%), while in the age group 80 +, there were more female than male deaths (58% vs. 42%). The leading triads of UCD, its consequence listed in Part 1 and CC listed in Part 2 are shown in Table 1. The leading UCD was ischemic heart disease, which was responsible for about 10% of total deaths in both age groups in 2019. On average, deaths with ischemic heart disease as UCD involved 2.55 additional causes, most frequently 3 causes of death. The leading non-UCD within the triads are typically organ failures, cardiac arrest, hypertensive disease or ischemic heart disease as well. These “chains “ are most often accompanied by other cardiovascular diseases (such as hypertension, congestive heart failure, or atrial fibrillation and flutter) or chronic conditions like diabetes mellitus, chronic obstructive pulmonary disease (COPD), chronic kidney disease, and dementia.

### Interplay between causes of death

For each of the leading triads of causes of death and for each model outlined in Fig. 1, we estimated arrival rates and derived the attributable fractions. Table 3 presents the pathway and causal pie model with the highest estimated attributable fraction, highlighting the most likely interaction scheme between diseases. The highest attributable fractions are mostly found in Model 2 with Pathway 5 or either Model 1 or Model 3 with Pathway 4. In both Models 1 and 3, the CC in Part 2 acts as a potential mediator in the disease chain outlined in Part 1 of the death certificate. In Pathway 4, it interacts with the conditions listed in Part 1 and mediates their relationship. In other words, in Model 1, this means that the UCD and the CC in Part 2 likely interact to cause the non-UCD in Part 1, whereas in Model 3, the interaction occurs between the CC in Part 2 and the non-UCD in Part 1.

**Table 3:**
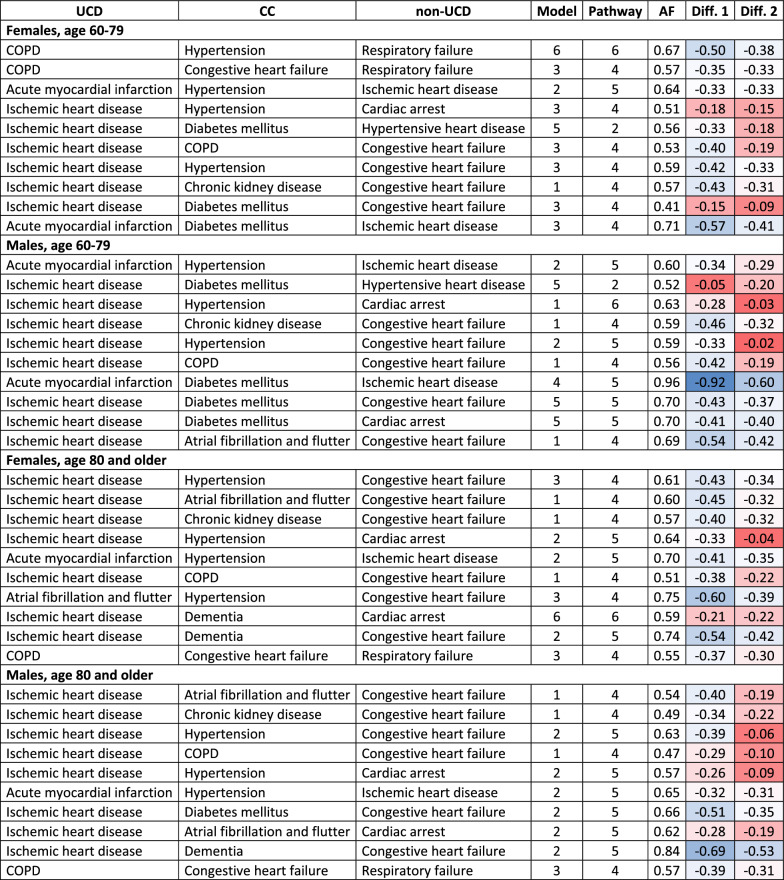
Causal pie models and pathways with the highest and second highest attributable fractions for leading triads of causes of death, by sex and age

Source: Authors'calculation

AF = attributable fraction of corresponding pathway and model; Diff. 1 = difference between the highest AF and the second highest AF in the same model; Diff. 2 = difference between the highest AF and the second highest AF across all models; In dark red, results with rather strong competing models and pathways are highlighted (diff. is lower than 10 pp)

Table 3 shows not only the scenarios with the highest attributable fractions but also the second highest, both within the same model and across all models. These estimates are presented as differences from the interpreted maxima. In most triads, these differences exceed at least 10 pp, indicating that the interpreted maxima are indeed strongly dominating mechanisms. However, in several triads, the differences are substantially lower. For example, in the triad chronic ischemic heart disease, diabetes and heart failure among younger females, Model 3 shows that the probability for leading Pathway 4 is only 15 pp higher than for Pathway 5 (see Table S3 in the Supplementary Material). This suggests that the interaction between E14 and I50 is relatively weaker in a substantial proportion of the population. Additionally, in this triad the attributable fraction is comparable with Model 2 Pathway 2 (diff. 9 pp), indicating that ischemic heart disease may moderate the relationship between diabetes and heart failure. Other rather inconclusive results are found in younger males in the triad of ischemic heart disease, diabetes and hypertensive disease. Although Model 5 has the highest AF, the proportion of individuals following Pathway 2 and Pathway 3 is comparable (see Table S3 in the Supplementary Material). The difference between these two pathways lies in whether a direct relationship between ischemic heart disease and hypertensive disease is presented, or whether it is mediated by diabetes. Lastly, strong competing models were found among (i) younger males in the triads ischemic heart disease and hypertension with either cardiac arrest and heart failure, (ii) older females in ischemic heart disease, hypertension and cardiac arrest and (iii) older males in the triad ischemic heart disease, hypertension and heart failure. In all previously mentioned triads, the highest AF values for Model 2 and Model 4 in Pathway 5 are comparable. This means, that it is rather unclear whether the UCD in these triads mediates the CC in Part 2 or non-UCD in Part 1.

In general, several patterns are seen in the roles of CC in Part 2. First, atrial fibrillation and flutter, heart failure, COPD, and chronic kidney disease predominantly act as mediators in the chains recorded in Part 1, as the highest AF were mostly found in Model 1, Pathway 4. This model is designed to reflect the interplay between causes of death in accordance with the WHO guidelines for cause of death certification. In contrast, the role of hypertension varies by age. Among premature deaths (under 80 years), it is an integral part of the lethal process (Model 3, Pathway 4). However, in deaths occurring at age 80 and above, hypertension remains outside the most probable mechanisms (Model 2, Pathway 5). We also included two triads involving dementia in the analysis. The results suggest that dementia did not play a mediating role. However, it was only examined in triads where it co-occurred with cardiovascular diseases. Lastly, diabetes covers the entire spectrum of possible roles in the lethal process, likely determined by both age at death and the accompanying conditions in the triads. It was identified to be an initiator of causal chains, although recorded as a CC in Part 2 (Model 5, Pathway 2), as well as a condition outside the primary mechanism (Model 5, Pathway 5), and as part of a strong synergy with the chain of morbid events (Model 3, Pathway 4).

## Discussion and conclusion

This study provided insight into the mechanism of synergy between the UCD, its consequent condition, and the CC in Part 2. Results suggested that CC in Part 2 are integral components of the trains of morbid events leading to death. This is because they can either mediate or even initiate these trains, and because there are significant differences in the lifespans of people who die from the same UCD, depending on whether any CC in Part 2 contributed to their death.

Three broader categories of roles that CCs in Part 2 can play in the lethal process can be distinguished. Some act as mediators in the chain of morbid events leading to death (atrial fibrillation and flutter, heart failure, COPD, chronic kidney disease), while others do not exhibit any interaction with the conditions listed in Part 1 (dementia). Additionally, as demonstrated by several models involving contributory diabetes, they can even play a role in the development of UCDs. Furthermore, for certain diseases—particularly hypertension and diabetes—the interplay between diseases might be age-dependent, with older age groups showing lower levels of synergy within triads.

On one hand, the mechanisms modelled in this study can be seen as an attempt to describe the interplay between diseases that, collectively, resulted in a single death. From this perspective, our contribution enhances the understanding of CC recorded in Part 2 of death certificates beyond their formal definition, which states “*other significant conditions that contributed to the fatal outcome, but were not related to the disease or condition directly causing death*” [47].

On the other hand, if we assume that mechanisms of dying are best described with medical knowledge and that death certificate data might not be detailed enough to do so, the findings can also be interpreted as an attempt to assess the accuracy of death certification—specifically, whether the practice aligns with WHO’s rules. From this perspective, we found that most triads were best fitted within the Model 1 Pathway 4, in which the UCD causes the non-UCD listed in Part 1 of the death certificate, with this relationship being moderated by the cause recorded in Part 2, or by Model 2 Pathway 5, which maintains the expected directional relationship between the UCD and the non-UCD in Part 1 but with CC not mediating this chain. Both of those mechanisms are in line with the WHO rules for causes of death certification. Although scenarios consistent WHO guidelines were the most common, in some cases, our results contradicted this logic. This was particularly evident in triads with dominating attributable fraction under Model 3 and Pathway 4. This mechanism essentially positions the synergy between the non-UCD in Part 1 and the CC in Part 2 as a prerequisite for the transition to the UCD—contradicting the definition of the UCD, which should represent the cause that initiates the chain of morbid events leading to death.

Apparently, Model 3 with Pathway 4 occurs especially in younger females and often involves organ failures and COPD. From a medical standpoint, this is an erroneous result, which could have several explanations. Firstly, this could be due to data issues. When working with multiple causes of death, it is almost impossible to differentiate between a'non-response'and the actual absence of the condition. Moreover, diseases vary in their susceptibility to non-reporting. It can be said that conditions which develop earlier in life and do not often lead directly to death (such as chronic diseases like COPD) are likely to have a higher probability of non-reporting compared to diseases that could be classified as immediate causes of death, like organ failures. This, of course, introduces biases into the underlying contingency tables used to estimate the model parameters, which could explain the reverse causation observed in Model 3.

Second, reverse causation could arise if important mediators are omitted from the modelled mechanisms. In this case, specific respiratory diseases might be missing from the chain between COPD and organ failure. These diseases may either not have been reported on the death certificate or represent a diverse group of conditions with a relatively low total number of deaths, leading to their exclusion from the analysis.

Both interpretations of the results contribute to filling the knowledge gap in multiple cause of death research, because (i) they differentiate multiple causes by the roles they played in death process and (ii) they focus on mechanisms rather than pairwise associations [22]. In addition to modelling disease synergy, the findings highlight the substantial heterogeneity in deaths that are otherwise hidden behind a single UCD in standard cause of death statistics. This is demonstrated, for example, by ischemic heart disease, which was the UCD for nearly one in ten deaths in the USA in 2019. However, we illustrated how the mechanisms leading to death classified as being caused by ischemic heart disease can vary. This became evident only through the implementation of multiple causes of death, which, in turn, enhance our understanding of the diversity in causes of death. Moreover, mortality is increasingly caused by the interactions between cardiovascular and respiratory diseases [48]. However, conventional mortality research, which focuses solely on UCD, inevitably disregards one of these conditions.

The study has several important limitations. First, questions regarding reliability of multiple causes of death (MCD) have been raised. Various studies have identified socio-economic factors that influence the reporting of MCD [49], while others have highlighted a lack of international standardization of MCD data [50, 51]. To determine whether our results apply beyond the USA, we replicated the analysis using Czech MCD data from 2018. The results indicated that, for each individual model, the pathways with the highest attributable fractions were consistent in both countries. However, we identified several differences in terms of the model with the absolute maximum AF. The differences were particularly evident in triads involving diabetes in younger age groups. This could be attributed to the overall variation in the completeness of medical data derived from death certificates, as reflected in the percentage of death certificates listing at least two causes of death. In Czechia, this percentage is significantly higher than in the USA (90% vs. 75%, respectively) and the differences in this regard are found across other countries too (in France 65% [32, 52], in Canada and Australia 80% [53, 54], in Sweden 60% [55]). Nevertheless, the differences between Czechia and the USA may once again highlight the variations in MCD between the two countries, warranting attention in future research.

Apart from the broader issue of data-related limitations, another one arises from the restricted interpretability of the results. For example, we found that older age groups tend to show lower levels of synergy between causes of death. This could be due to the overall lower morbidity among individuals who survive to older ages, where lower morbidity might be a necessary condition for reaching advanced age. Alternatively, the way diseases are recorded may change with age. In the oldest age groups, death certifiers might be more inclined to record causes of death primarily as parts of causal chains rather than as CC in Part 2. This is supported by the fact that in the USA, the decline in the average number of diseases reported per death certificate is slightly steeper when considering only CC in Part 2, compared to the decline in conditions reported as part of the chains of morbid events leading to death, as shown in the Supplementary Material.

Other limitations stem from the methodological framework used, that is based on several assumptions, which are summarized in the method section. However, these assumptions may be far from reality. For example, the assumption of monotonicity—that the presence of diseases can only increase the probability of transitioning to a terminal state—may not fully apply in MCD. This is partly due to socio-demographically conditioned recording of MCD, which can ultimately manifest as missing-not-at-random data. Secondly, the way the risk of death changes with the progression of morbid chains is not yet well understood. Another limitation is that the modelled mechanisms are restricted to only three diseases, whereas many more could have been present at the time of death, and some of these may not even have been recorded as causes of death.

To conclude, we found that contributory causes of death are mediators of chains of morbid events leading to death and therefore they play a crucial role in transitions to terminal morbid states. Therefore, considering multiple causes of death is a way to understand diversity among deaths from the same underlying causes.

## Supplementary Information

Additional file 1.

## Data Availability

Data are publicly available at: https://www.cdc.gov/nchs/nvss/mortality_public_use_data.htm.
