# Supplementary material for: Are contributory causes of death in part 2 of the death certificate mediators of chains of morbid events leading to death?
Source: Popul Health Metr. 2025 Aug 7;23:46. doi: 10.1186/s12963-025-00394-w (PMC12333147; doi:10.1186/s12963-025-00394-w)
Supplement: Supplementary file 1 — Additional file 1. [file 12963_2025_394_MOESM1_ESM.docx]

**Supplementary material**

1. **ICD-10 codes and corresponding causes of death from the selected triads of diseases**

*Table S1: ICD-10 codes used in the analysis*

| **ICD-10 code** | **Cause of death** |
| --- | --- |
| I13 | Hypertensive heart and renal disease |
| I21 | Acute myocardial infarction |
| I25 | Chronic ischemic heart disease (ischemic heart disease) |
| I48 | Atrial fibrillation and flutter |
| J44 | Chronic obstructive pulmonary disease (COPD) |
| I10 | Essential (primary) hypertension (hypertension) |
| I11 | Hypertensive heart disease |
| I46 | Cardiac arrest |
| I50 | Congestive heart failure |
| J96 | Respiratory failure |
| N18 | Chronic kidney disease |
| E14 | Diabetes mellitus (diabetes) |
| F03 | Dementia |
| G30 | Alzheimer's disease |

1. **Pathways for models 2, 3, 4, 5 and 6**

Model 2

I The CC causes the non-UCD directly (path D2).

II The CC causes the UCD, which in turn causes the non-UCD (path M1D3).

III The CC causes the UCD, and then both interact to cause the non-UCD (path M1D4).

IV The CC and an exogenous mediator interact to cause the non-UCD (path M2D4).

V An exogenous mediator causes the non-UCD directly (path M2D3).

VI Neither the CC nor the UCD causes the non-UCD (path D1).

Model 3

I The non-UCD causes the UCD directly (path D2).

II The non-UCD causes the CC, which in turn causes the UCD (path M1D3).

III The non-UCD causes the CC, and then both interact to cause the UCD (path M1D4).

IV The non-UCD and an exogenous mediator interact to cause the UCD (path M2D4).

V An exogenous mediator causes the UCD directly (path M2D3).

VI Neither the non-UCD nor the CC causes the UCD (path D1).

Model 4

I The non-UCD causes the CC directly (path D2).

II The non-UCD causes the UCD, which in turn causes the CC (path M1D3).

III The non-UCD causes the UCD, and then both interact to cause the CC (path M1D4).

IV The non-UCD and an exogenous mediator interact to cause the CC (path M2D4).

V An exogenous mediator causes the CC directly (path M2D3).

VI Neither the non-UCD nor the UCD causes the CC (path D1).

Model 5

I The CC causes the UCD directly (path D2).

II The CC causes the non-MCD, which in turn causes the UCD (path M1D3).

III The CC causes the non-UCD, and then both interact to cause the UCD (path M1D4).

IV The CC and an exogenous mediator interact to cause the UCD (path M2D4).

V An exogenous mediator causes the UCD directly (path M2D3).

VI Neither the CC nor the non-UCD causes the UCD (path D1).

Model 6

I The UCD causes the CC directly (path D2).

II The UCD causes the non-UCD, which in turn causes the CC (path M1D3).

III The UCD causes the non-UCD, and then both interact to cause the CC (path M1D4).

IV The UCD and an exogenous mediator interact to cause the CC (path M2D4).

V An exogenous mediator causes the CC directly (path M2D3).

VI Neither the UCD nor the non-UCD causes the CC (path D1).

1. **Example of the distribution of deaths in Table 2**

Triad: Chronic ischemic heart disease (UCD), Atrial fibrillation and flutter (CC in Part 2) and Congestive heart failure (non-UCD in Part 1), Males, 80+

*Table S2: Example of the distribution of deaths in contingency tables*

| **Model 1** | | | |  | **Model 2** | | | |  | **Model 3** | | | |
| --- | --- | --- | --- | --- | --- | --- | --- | --- | --- | --- | --- | --- | --- |
| M | D | E | |  | M | D | E | |  | M | D | E | |
| CC | non-UCD | UCD=0 | UCD=1 |  | UCD | non-UCD | CC=0 | CC=1 |  | CC | UCD | non-UCD=0 | non-UCD=1 |
| 0 | 0 | 240368 | 40060 |  | 0 | 0 | 240368 | 23227 |  | 0 | 0 | 240368 | 33694 |
| 1 | 0 | 23227 | 3056 |  | 1 | 0 | 40060 | 3056 |  | 1 | 0 | 23227 | 3710 |
| 0 | 1 | 33694 | 16840 |  | 0 | 1 | 33694 | 3710 |  | 0 | 1 | 40060 | 16840 |
| 1 | 1 | 3710 | 3143 |  | 1 | 1 | 16840 | 3143 |  | 1 | 1 | 3056 | 3143 |
| **Model 4** | | | |  | **Model 5** | | | |  | **Model 6** | | | |
| M | D | E | |  | M | D | E | |  | M | D | E | |
| UCD | CC | non-UCD=0 | non-UCD=1 |  | non-UCD | UCD | CC=0 | CC=1 |  | non-UCD | CC | UCD=0 | UCD=1 |
| 0 | 0 | 240368 | 33694 |  | 0 | 0 | 240368 | 23227 |  | 0 | 0 | 240368 | 40060 |
| 1 | 0 | 40060 | 16840 |  | 1 | 0 | 33694 | 3710 |  | 1 | 0 | 33694 | 16840 |
| 0 | 1 | 23227 | 3710 |  | 0 | 1 | 40060 | 3056 |  | 0 | 1 | 23227 | 3056 |
| 1 | 1 | 3056 | 3143 |  | 1 | 1 | 16840 | 3143 |  | 1 | 1 | 3710 | 3143 |

1. **Technical details of the method**

The parameters of the causal pie model, the vector of six lambdas, represent the arrival rates of specific subpopulations into the observed states. Their raw estimates can be computed directly from the data using the system of the equations with the notation from table (Chen & Lee, 2018):

$\lambda_{M1}=-\frac{1}{T}\times ln\left( 1-\frac{m_{2}+m_{4}}{m_{1}+m_{2}+m_{3}+m_{4}} \right)$ (1)

$\lambda_{M2}=-\frac{1}{T}\times ln\left( 1-\frac{n_{2}+n_{4}}{n_{1}+n_{2}+n_{3}+n_{4}} \right)-\lambda_{M1}$ (2)

$\lambda_{D1}=-\frac{1}{T}\times ln\left( 1-\frac{m_{3}}{m_{1}+m_{2}+m_{3}+m_{4}} \right)$ (3)

$\lambda_{D2}=-\frac{1}{T}\times ln\left( 1-\frac{n_{3}}{n_{1}+n_{2}+n_{3}+n_{4}} \right)-\lambda_{D1}$ (4)

$\lambda_{D3}=\frac{m_{4}}{(m_{2}+m_{4})\times(T-ET1)}-\lambda_{D1}$ (5)

$\lambda_{D3}=\frac{n_{4}}{(n_{2}+n_{4})\times(T-ET2)}-\lambda_{D1}-\lambda_{D2}-\lambda_{D3}$ (6)

Where ET1 equals to:

$ET1=\frac{\frac{1}{\lambda_{M1}}-(T+\frac{1}{\lambda_{M1}})\times e^{-\lambda_{M1}\times T}}{1-e^{-\lambda_{M1}\times T}}$ (7)

And ET2 equals to:

$ET2=\frac{\frac{1}{\lambda_{M1}+\lambda_{M2}}-(T+\frac{1}{\lambda_{M1}+\lambda_{M2}})\times e^{-(\lambda_{M1}+\lambda_{M2})\times T}}{1-e^{-(\lambda_{M1}+\lambda_{M2})\times T}}$ (8)

The m_i_ and n_i_ correspond to the death counts depending on the cross classification used for each model. As an example, see the distribution of death by causes in triad chronic ischemic heart disease (UCD), atrial fibrillation and flutter (CC in Part 2) and congestive heart failure (non-UCD in Part 1) (Males, 80+) above.

Using the example data shown in the table above, $\lambda_{M1}$for Model 1 can be estimated as:

$$\lambda_{M1}=-\frac{1}{1}\times ln\left( 1-\frac{23 227+3 710}{240 368+23 227+33 694+3 710} \right)=0,09375$$

The raw estimate of the arrival rate along the M1 edge is 0.09375 (~9.4%).

To find the maximum likelihood estimates of the causal pie parameters, the expression is maximized:

$l=\sum_{i=1}^{4} m_{i}\times\log\left( p_{0i} \right)+\sum_{i=1}^{4} n_{j}\times\log\left( p_{1j} \right)$ (9)

Where p_0i_ and p_1j_ are probabilities of transition between exposure and outcome for exposed population and unexposed population respectively. The probabilities are converted from the arrival rates using formulas (Richiardi et al., 2013; Chen & Lee, 2018):

$p_{01}=e^{-(\lambda_{M1}+\lambda_{D1})\times T}$ (10)

$p_{02}=\frac{\lambda_{M1}}{\lambda_{D3-}\lambda_{M1}}\times\left[ e^{-(\lambda_{M1}+\lambda_{D1})\times T}-e^{-(\lambda_{D1}+\lambda_{D3})\times T} \right]$ (11)

$p_{03}=\frac{\lambda_{D1}}{\lambda_{M1+}\lambda_{D1}}\times\left[ 1-e^{-(\lambda_{M1}+\lambda_{D1})\times T} \right]$ (12)

$p_{04}=1-p_{01}-p_{02}-p_{03}$ (13)

$p_{11}=e^{-(\lambda_{M1}+\lambda_{M2}+\lambda_{D1+}\lambda_{D2})\times T}$ (14)

$p_{12}=\frac{\lambda_{M1}+\lambda_{M2}}{\lambda_{D3}+\lambda_{D4}-{(\lambda}_{M1}+\lambda_{M2})}\times\left[ e^{-(\lambda_{M1}+\lambda_{M2}+\lambda_{D1}+\lambda_{D2})\times T}-e^{-(\lambda_{D1}+\lambda_{D2}+\lambda_{D3}+\lambda_{D4})\times T} \right]$ (15)

$p_{13}=\frac{\lambda_{D1}+\lambda_{D2}}{\lambda_{D1}+\lambda_{D2}+\lambda_{M1}+\lambda_{M2}}\times\left[ {1-e}^{-(\lambda_{M1}+\lambda_{M2}+\lambda_{D1}+\lambda_{D2})\times T} \right]$ (16)

$p_{14}=1-p_{11}-p_{12}-p_{13}$ (17)

Chen & Lee (2018) suggest solving the optimization of maximum likelihood function numerically, by implementing the Newton-Rapson algorithm. The NR algorithm is a way to find roots of systems of equations (Jennrich & Robinson, 1969; McCulloh, 1997). The likelihood function is differentiated in respect to lambda parameters iteratively (Jennrich & Robinson, 1969; McCulloh, 1997). After each iteration, the increase in likelihood is evaluated and once the sufficient increase is not achieved, the algorithm stops and returns its latest input parameters (Ypma, 1995). The input parameters for the NR algorithm are the vector of lambdas that can be directly computed from the data applying the formulas 1-6 (Chen & Lee, 2018).

After finding the maximum likelihood estimators of arrival rates, the attributable fractions are calculated with the logic of backward attribution of the outcome. Chen & Lee (2018) uses equations to determine the probability for each of the six pathways (attributable fractions):

${AF}_{I}=\frac{n_{4}}{n_{3}+n_{4}+m_{3}+m_{4}}\times\frac{\lambda_{D2}}{\lambda_{D1}+\lambda_{D2}+\lambda_{D3}+\lambda_{D4}}+\frac{n_{3}}{n_{3}+n_{4}+m_{3}+m_{4}}\times\frac{\lambda_{D2}}{\lambda_{D1}+\lambda_{D2}}$ (18)

${AF}_{II}=\frac{n_{4}}{n_{3}+n_{4}+m_{3}+m_{4}}\times\frac{\lambda_{D3}}{\lambda_{D1}+\lambda_{D2}+\lambda_{D3}+\lambda_{D4}}\times\frac{\lambda_{M2}}{\lambda_{M1}+\lambda_{M2}}$ (19)

${AF}_{III}=\frac{n_{4}}{n_{3}+n_{4}+m_{3}+m_{4}}\times\frac{\lambda_{D4}}{\lambda_{D1}+\lambda_{D2}+\lambda_{D3}+\lambda_{D4}}\times\frac{\lambda_{M2}}{\lambda_{M1}+\lambda_{M2}}$ (20)

${AF}_{IV}=\frac{n_{4}}{n_{3}+n_{4}+m_{3}+m_{4}}\times\frac{\lambda_{D4}}{\lambda_{D1}+\lambda_{D2}+\lambda_{D3}+\lambda_{D4}}\times\frac{\lambda_{M1}}{\lambda_{M1}+\lambda_{M2}}$ (21)

${AF}_{V}=\frac{n_{4}}{n_{3}+n_{4}+m_{3}+m_{4}}\times\frac{\lambda_{D3}}{\lambda_{D1}+\lambda_{D2}+\lambda_{D3}+\lambda_{D4}}\times\frac{\lambda_{M1}}{\lambda_{M1}+\lambda_{M2}}+\frac{m_{4}}{n_{3}+n_{4}+m_{3}+m_{4}}\times\frac{\lambda_{D3}}{\lambda_{D1}+\lambda_{D3}}$ (22)

${AF}_{VI}=\frac{n_{4}}{n_{3}+n_{4}+m_{3}+m_{4}}\times\frac{\lambda_{D1}}{\lambda_{D1}+\lambda_{D2}+\lambda_{D3}+\lambda_{D4}}+\frac{n_{3}}{n_{3}+n_{4}+m_{3}+m_{4}}\times\frac{\lambda_{D1}}{\lambda_{D1}+\lambda_{D2}}+\frac{m_{4}}{n_{3}+n_{4}+m_{3}+m_{4}}\times\frac{\lambda_{D1}}{\lambda_{D1}+\lambda_{D2}}+\frac{m_{3}}{n_{3}+n_{4}+m_{3}+m_{4}}$ (23)

1. **Average number of conditions per death certificate**

*
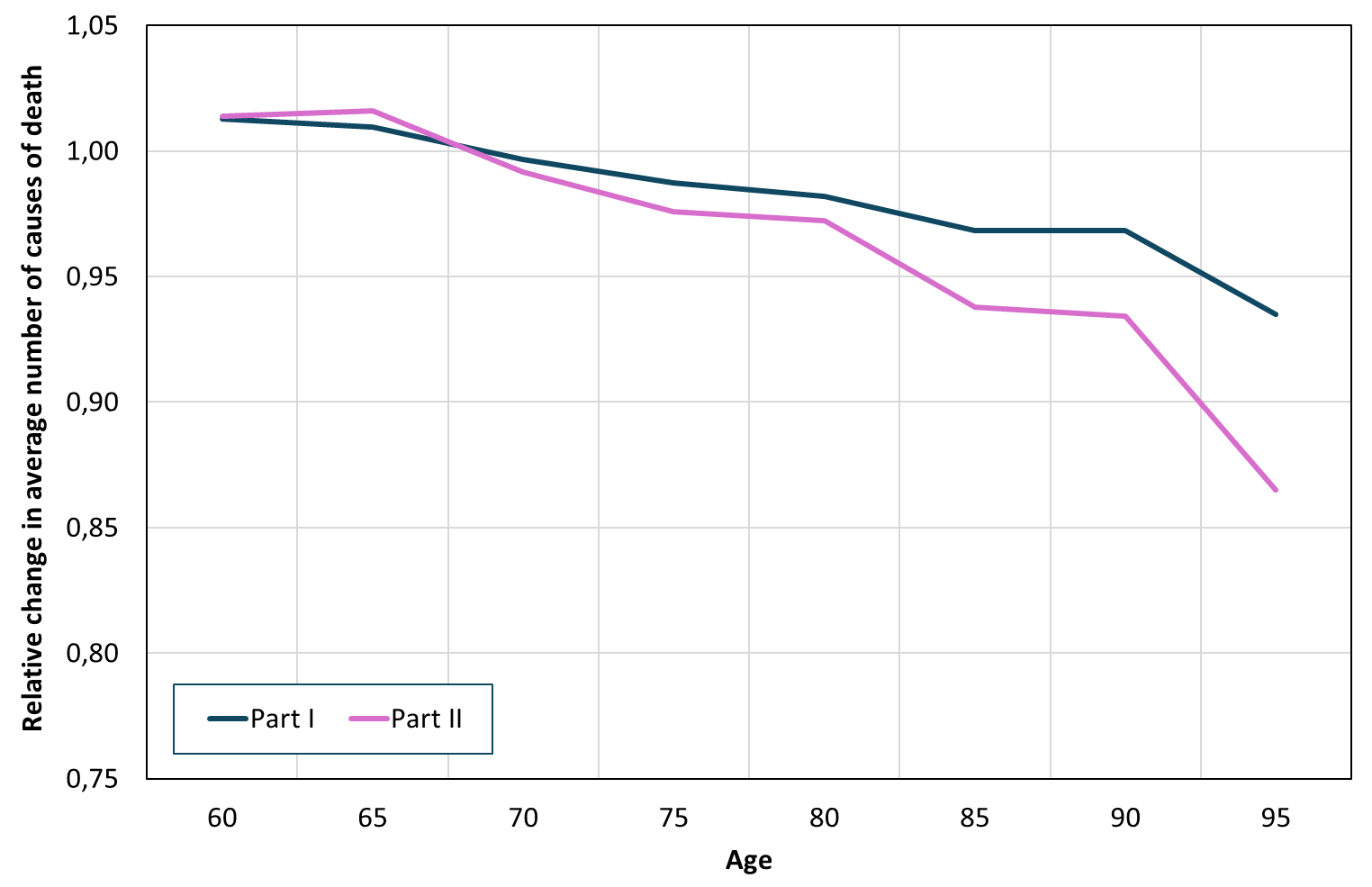
Figure : Relative changes in average number of conditions per death certificate by age and types of causes of death, USA, 2019*

The figure shows changes in the average number of conditions reported by the death certifier by age compared to the previous age group. The average is computed separately for conditions recorded in Part 1 of the death certificate (blue line) and Part 2 (pink line). For example, the starting value of 1.01 for both lines indicates that, at age 60, the average number of diseases reported in both parts of the death certificate was 1 percentage point higher compared to age 55. The figure illustrates a steeper decline in the average number of conditions recorded in Part 2, compared to Part 1. This may suggest that at older ages, physicians tend to record more diseases in Part 1, considering them as conditions directly related to the underlying cause, which could be a limitation in the causal pie analysis.

1. **Extended table 4 (attributable fractions)**

*Table S3: Causal pie models and pathways with the highest and second highest attributable fractions for leading triads of causes of death, by sex and age*

| Disease triad and subpopulation | Highest AF | | | Second highest in the same model | | | Difference | Second highest across all models | | |  |
| --- | --- | --- | --- | --- | --- | --- | --- | --- | --- | --- | --- |
|  | Model | Pathway | AF1 | Model | Pathway | AF2 | AF1-AF2 | Model | Pathway | AF3 | AF1-AF3 |
| F_1_J44_I10_J96 | 6 | VI | 0.67 | 6 | IV | 0.17 | 0.50 | 4 | IV | 0.29 | 0.38 |
| F_1_J44_I50_J96 | 3 | IV | 0.57 | 3 | V | 0.22 | 0.35 | 6 | VI | 0.24 | 0.33 |
| F_1_I21_I10_I25 | 2 | V | 0.64 | 2 | IV | 0.31 | 0.33 | 3 | I | 0.31 | 0.33 |
| F_1_I25_I10_I46 | 3 | IV | 0.51 | 3 | VI | 0.33 | 0.18 | 2 | VI | 0.36 | 0.15 |
| F_1_I25_E14_I11 | 5 | II | 0.56 | 5 | VI | 0.23 | 0.33 | 1 | III | 0.38 | 0.18 |
| F_1_I25_J44_I50 | 3 | IV | 0.53 | 3 | I | 0.13 | 0.40 | 4 | V | 0.34 | 0.19 |
| F_1_I25_I10_I50 | 3 | IV | 0.59 | 3 | I | 0.17 | 0.42 | 2 | IV | 0.25 | 0.33 |
| F_1_I25_N18_I50 | 1 | IV | 0.57 | 1 | V | 0.14 | 0.43 | 5 | IV | 0.26 | 0.31 |
| F_1_I25_E14_I50 | 3 | IV | 0.41 | 3 | V | 0.25 | 0.15 | 2 | II | 0.32 | 0.09 |
| F_1_I21_E14_I25 | 3 | IV | 0.71 | 3 | I | 0.14 | 0.57 | 5 | VI | 0.30 | 0.41 |
| M_1_I21_I10_I25 | 2 | V | 0.60 | 2 | IV | 0.26 | 0.34 | 4 | IV | 0.31 | 0.29 |
| M_1_I25_E14_I11 | 5 | II | 0.52 | 5 | III | 0.47 | 0.05 | 1 | III | 0.32 | 0.20 |
| M_1_I25_I10_I46 | 1 | VI | 0.63 | 1 | V | 0.35 | 0.28 | 3 | VI | 0.60 | 0.03 |
| M_1_I25_N18_I50 | 1 | IV | 0.59 | 1 | V | 0.14 | 0.46 | 5 | IV | 0.27 | 0.32 |
| M_1_I25_I10_I50 | 2 | V | 0.59 | 2 | IV | 0.26 | 0.33 | 4 | V | 0.57 | 0.02 |
| M_1_I25_J44_I50 | 1 | IV | 0.56 | 1 | I | 0.14 | 0.42 | 4 | V | 0.37 | 0.19 |
| M_1_I21_E14_I25 | 4 | V | 0.96 | 4 | VI | 0.04 | 0.92 | 6 | II | 0.36 | 0.60 |
| M_1_I25_E14_I50 | 5 | V | 0.70 | 5 | VI | 0.27 | 0.43 | 4 | IV | 0.33 | 0.37 |
| M_1_I25_E14_I46 | 5 | V | 0.70 | 5 | VI | 0.29 | 0.41 | 6 | IV | 0.30 | 0.40 |
| M_1_I25_I48_I50 | 1 | IV | 0.69 | 1 | V | 0.14 | 0.54 | 5 | IV | 0.27 | 0.42 |
| F_2_I25_I10_I50 | 3 | IV | 0.61 | 3 | I | 0.18 | 0.43 | 2 | IV | 0.28 | 0.34 |
| F_2_I25_I48_I50 | 1 | IV | 0.60 | 1 | V | 0.15 | 0.45 | 5 | VI | 0.28 | 0.32 |
| F_2_I25_N18_I50 | 1 | IV | 0.57 | 1 | V | 0.17 | 0.40 | 3 | III | 0.25 | 0.32 |
| F_2_I25_I10_I46 | 2 | V | 0.64 | 2 | VI | 0.31 | 0.33 | 4 | V | 0.60 | 0.04 |
| F_2_I21_I10_I25 | 2 | V | 0.70 | 2 | IV | 0.28 | 0.41 | 6 | III | 0.35 | 0.35 |
| F_2_I25_J44_I50 | 1 | IV | 0.51 | 1 | I | 0.14 | 0.38 | 4 | IV | 0.30 | 0.22 |
| F_2_I48_I10_I50 | 3 | IV | 0.75 | 3 | I | 0.15 | 0.60 | 4 | V | 0.36 | 0.39 |
| F_2_I25_F03_I46 | 6 | VI | 0.59 | 6 | V | 0.38 | 0.21 | 4 | V | 0.38 | 0.22 |
| F_2_I25_F03_I50 | 2 | V | 0.74 | 2 | VI | 0.20 | 0.54 | 5 | II | 0.32 | 0.42 |
| F_2_J44_I50_J96 | 3 | IV | 0.55 | 3 | V | 0.18 | 0.37 | 1 | III | 0.26 | 0.30 |
| M_2_I25_I48_I50 | 1 | IV | 0.54 | 1 | I | 0.14 | 0.40 | 2 | V | 0.35 | 0.19 |
| M_2_I25_N18_I50 | 1 | IV | 0.49 | 1 | III | 0.15 | 0.34 | 3 | III | 0.27 | 0.22 |
| M_2_I25_I10_I50 | 2 | V | 0.63 | 2 | IV | 0.24 | 0.39 | 4 | V | 0.58 | 0.06 |
| M_2_I25_J44_I50 | 1 | IV | 0.47 | 1 | I | 0.18 | 0.29 | 2 | IV | 0.37 | 0.10 |
| M_2_I25_I10_I46 | 2 | V | 0.57 | 2 | VI | 0.30 | 0.26 | 4 | V | 0.48 | 0.09 |
| M_2_I21_I10_I25 | 2 | V | 0.65 | 2 | IV | 0.33 | 0.32 | 6 | III | 0.33 | 0.31 |
| M_2_I25_E14_I50 | 2 | V | 0.66 | 2 | VI | 0.15 | 0.51 | 4 | IV | 0.31 | 0.35 |
| M_2_I25_I48_I46 | 2 | V | 0.62 | 2 | VI | 0.34 | 0.28 | 5 | V | 0.44 | 0.19 |
| M_2_I25_F03_I50 | 2 | V | 0.84 | 2 | VI | 0.16 | 0.69 | 1 | I | 0.32 | 0.53 |
| M_2_J44_I50_J96 | 3 | IV | 0.57 | 3 | V | 0.17 | 0.39 | 6 | IV | 0.25 | 0.31 |

Note: F_2 = females, age 80+; F_1 = females, age 60-79; M_2 = males, age 80+; M_1 = males, age 60-79

1. **References used in Supplementary file**

Richiardi, L., Bellocco, R., & Zugna, D. (2013). Mediation analysis in epidemiology: methods, interpretation and bias. *International journal of epidemiology*, *42*(5), 1511-1519.

Chen, C., & Lee, W. C. (2018). Attributing diseases to multiple pathways: a causal-pie modeling approach. *Clinical epidemiology*, 489-497.

Jennrich, R. I., & Robinson, S. M. (1969). A Newton-Raphson algorithm for maximum likelihood factor analysis. *Psychometrika*, *34*(1), 111-123.

McCulloch, C. E. (1997). Maximum likelihood algorithms for generalized linear mixed models. Journal of the American statistical Association, 92(437), 162-170.

Ypma, T. J. (1995). Historical development of the Newton–Raphson method. *SIAM review*, *37*(4), 531-551.
